# Effects of the performance parameters of a wheelchair on the changes in the position of the centre of gravity of the human body in dynamic condition

**DOI:** 10.1371/journal.pone.0226013

**Published:** 2019-12-06

**Authors:** Bartosz Wieczorek, Mateusz Kukla

**Affiliations:** Department of Basics Machine Design, Poznan University of Technology, Poznan, Poland; University of Illinois at Urbana-Champaign, UNITED STATES

## Abstract

**Purpose:**

The aim of this research is to establish whether, and to what extent, the tilt angle, gear ratio of the propulsion system and propulsion frequency of a wheelchair influence the position of the centre of gravity. Furthermore, it verifies the usefulness of such research using an original test stand.

**Materials and methods:**

The article presents the effects of three operational parameters of a wheelchair on the position of the centre of gravity of the human body. The study included 27 wheelchair propulsion tests of a wheelchair with pushrim propulsion using the following variable parameters: gear ratio of the propulsion system, propulsion frequency and wheelchair tilt angle. The position of the centre of gravity of the human body was measured in dynamic conditions at 100 Hz. The results were represented with ellipses defining the region of variability of the position of the centre of gravity of the human body. The coordinates of the centre of gravity were measured in relation to the reference system, with the start point at the centre of the axis of rotation of the rear wheelchair wheels. The measurements were taken in a horizontal plane in relation to the base on which the test stand was positioned.

**Results:**

The research carried out shows that the inclination angle of the wheelchair has the greatest influence on position of the ellipse describing the position of the centre of gravity of the human body. By controlling the change in the inclination angle value in the range from 0° to 5.4°, the standard deviation of the length of the horizontal half-axis of the ellipse (SD a) equal to 31.2 mm was obtained. For comparison, by changing the frequency of pushes (40 to 50 pushes per minute) of the wheelchair at a constant inclination angle, the standard deviation of the horizontal half-axis length (SD a) equal to 8 mm was recorded. The results of the study show a change in the position of the centre of gravity of the human body in dynamic conditions. They are relative to the contact points of the wheelchair wheels with the ground. Using the dimensions of the plotted ellipses, one can determine the values of pressure that affect the wheelchair’s individual wheels. Conclusions–Increasing the value of each aforementioned parameter resulted in the increase of strength required by the operator to propel the wheelchair. It directly influenced the position of the centre of gravity during the test.

## Introduction

The operating conditions of the propulsion system [1, 2] have a significant effect on the biomechanical parameters [3] of manual wheelchairs, including the position of the centre of gravity of the human body. Manual wheelchairs can be self-propelled and require the user to use their upper limbs to move the wheelchair. A commonly used manual propulsion system is a pushrim propulsion system, in which the user independently propels the left and the right wheel by pushing round pushrims attached to the rim of each wheel. It requires movement of the upper limb [4], and depending on the degree of disability, may also require use of a shoulder girdle [5]. As a result, the position of the centre of gravity of the anthropotechnical system [6, 7] changes. An anthropotechnical system is a system that involves a human user as well as a technical device in order to function. An example of a mutually related anthropotechnical system is a man-wheelchair system.

The nature of the manual propulsion system requires the use of the muscular system [8] by the disabled person, which in turn, may result in fatigue [9]. Fatigue depends on the effort required, and is affected by the resistance to motion [10, 11] and the propulsion frequency. Multi-gear transmissions [12, 13] can be used to reduce the load on the muscular system as a result of the operation of the manual propulsion system.

Apart from the technical solutions reducing the propulsion-related loads, a disabled person can further reduce it by maintaining correct body kinematics. Depending on the operational parameters of a wheelchair, kinematics of the body segments propelling the wheelchair also change. According to various studies, the propulsion frequency is affected by the flexion angles of the joints of the upper limb [14]. Analysis of the kinematics of the body of the operator propelling the wheelchair also shows the effect of resistance to motion on torso flexion [15]. When using the wheelchair on slopes, the position of the body shifts in relation to the rear drive axis [2], the center of pressure displacement for the seat is also changing [26]. In this case, the effect is the same as when adjusting the wheelchair seat, however, the changes are dynamic in nature. As a result, the flexion angle of the upper limb segments [16] changes to compensate the change in position in relation to the axis of rotation of the drive wheel. Also, the wheelchair tilt angle shifts the position of the centre of gravity of the human body forwards or backwards depending on the slope angle.

Based on the previous analyses, the locomotive function relying on the upper limbs of the user depends on the operational parameters of the wheelchair. Different operational parameters can affect the kinematics of the human body. Any changes in the kinematics of the human body segments will translate into changes in the position of its centre of gravity. Observations show that the selected operational parameters of the wheelchair affect the position of the centre of gravity of the human body. The operational parameters including the wheelchair tilt angle, gear ratio of the propulsion system and the propulsion frequency are the main components of the locomotive function. The position of the centre of gravity of the human body as a whole depends on how its individual segments are oriented in space. It is therefore reasonable to analyse how the wheelchair’s operational parameters affect the centre of gravity, but also how it changes its position in dynamic conditions related to manual propelling. Determination of changes in the centre of gravity is important, because it allows to determine the place of application of the load resulting from human body weight. In addition, the position of the centre of gravity of the human is important when determining the stability of the wheelchair, both static and dynamic.

The study of the impact of operational parameters required the use of a specialised test stand that allows the measurement of dynamic [18] and biomechanical parameters. It also allows simultaneous simulation of various operational parameters of the wheelchair [19]. The test stand was designed and manufactured as part of the Lider VII project “Study of the biomechanics of manually propelled wheelchair for innovative manual and hybrid drives” (LIDER/7/0025/L-7/15/2016) financed by the National Centre for Research and Development. The aim of this research is to establish whether, and to what extent, the tilt angle, gear ratio of the propulsion system and propulsion frequency of a wheelchair influence the position of the centre of gravity. Furthermore, it verifies the usefulness of such research using an original test stand.

## Materials and methods

The method of determining the position of the centre of gravity can be divided into three stages: preparation for the test, the measurement test and the analytical processing of the obtained data. As part of the preparations, the wheelchair was weighed, then along with the examined person, it was placed on the test bench. After securing the wheelchair, its position was measured to determine the exact position relative to the reference system adopted in the study. The examined person performed 30 propulsion cycles with a constant frequency (equal time intervals between pushes). Each measurement test was carried out with its unique and constant operational parameters, which include: the frequency of pushes, the inclination of the wheelchair and the gear ratio.

**Fig 1:**
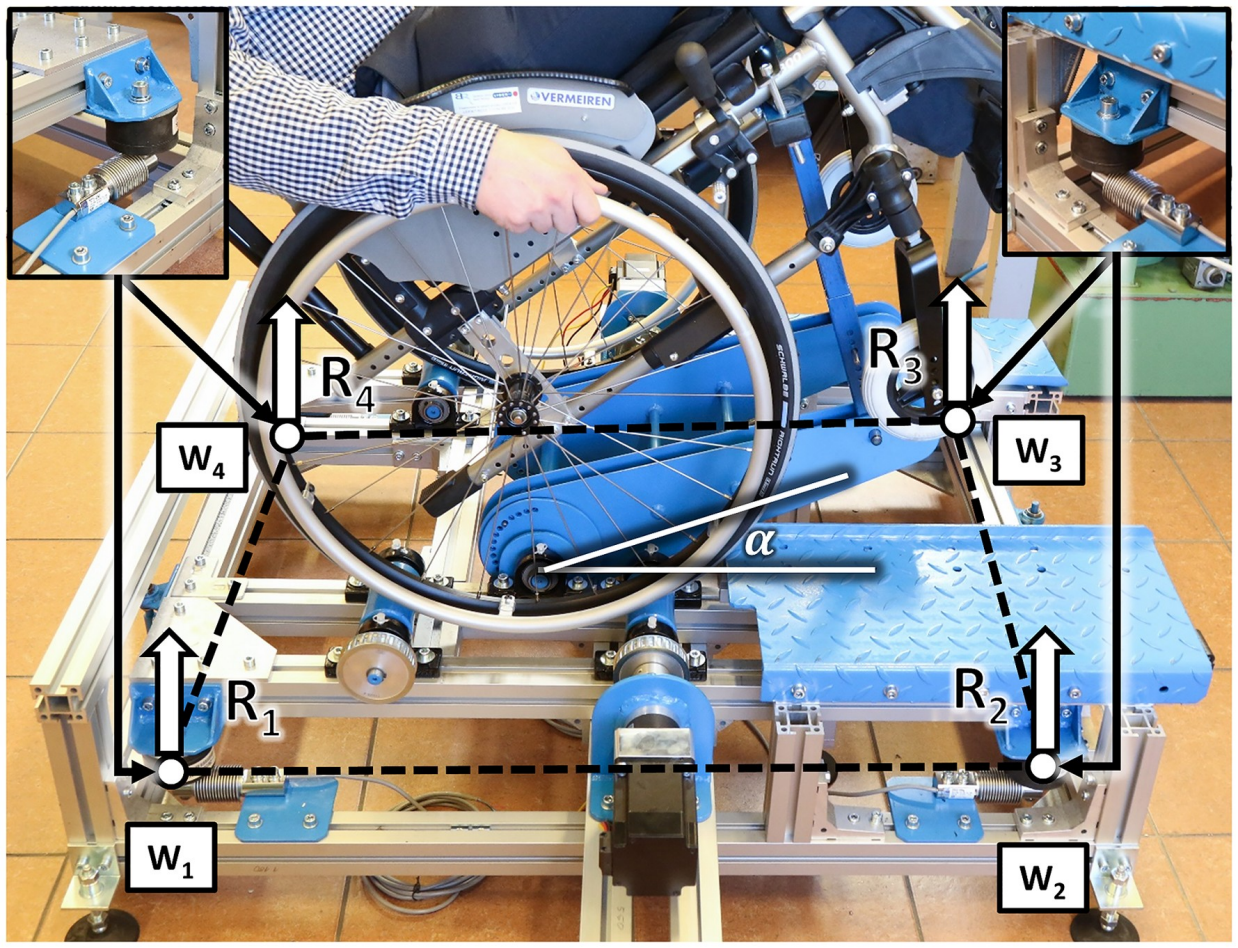
Test stand with strain gauges W_1_–W_4_ and the reaction force at the supports of the weighing scale platform R_1_–R_4_.

The wheelchair was tested using a wheelchair dynamometer [17] with a weighing scale with four strain gauges W_i_ (Fig 1). The weighing scale was used to determine the response in the supports of the weighing scale platform on which the wheelchair and its operator were positioned. The wheelchair on the weighing scale platform can be propelled by the operator and a wheel torque is compensated by the traction rollers in the weighing scale platform.

**Fig 2:**
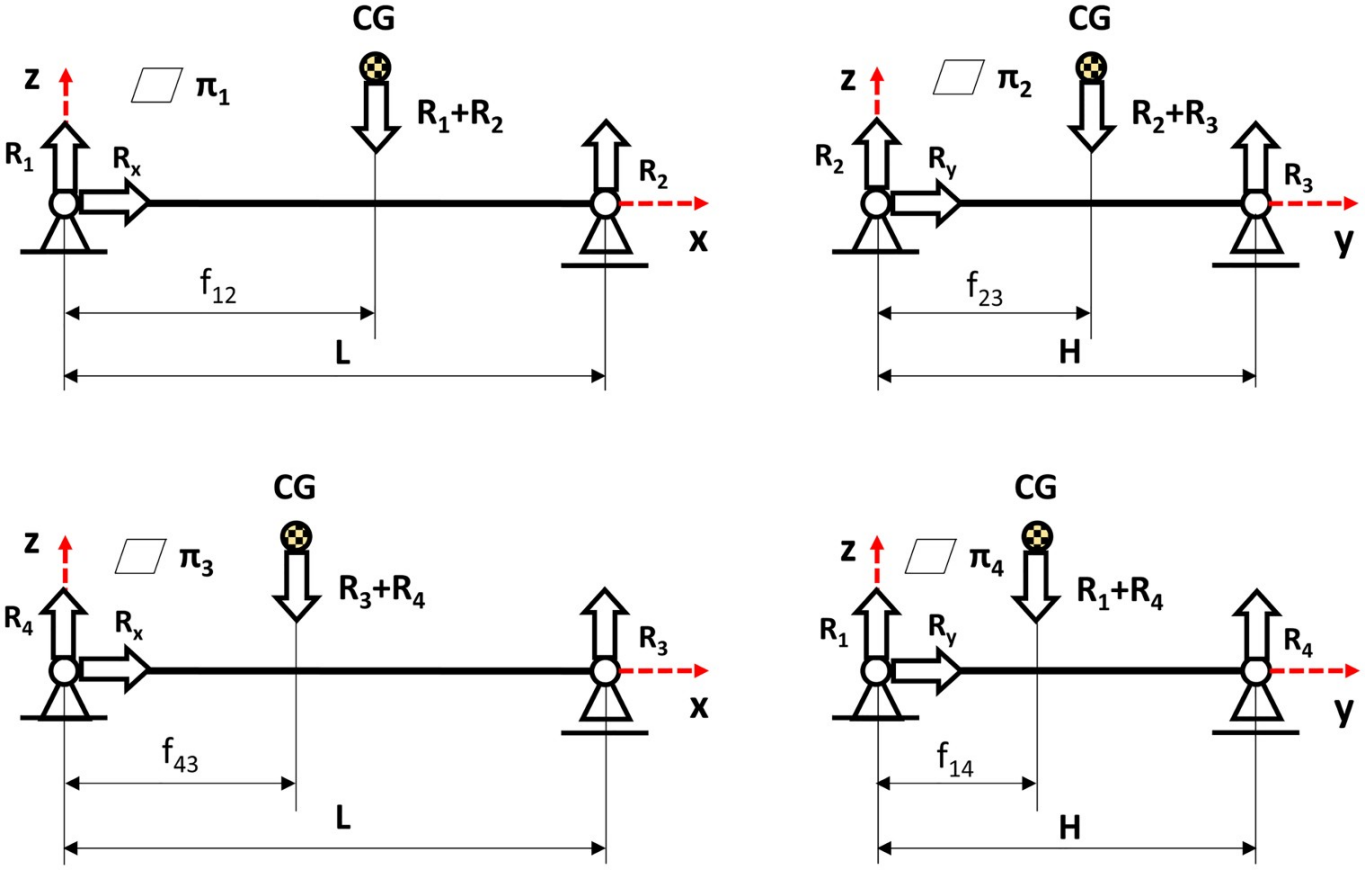
Diagram including data for determining the position of the centre of gravity, where: CG—Centre of gravity, πi—One of four side planes, Ri—Reaction of the support of the weighing scale platform, f_ij_−position of the centre of gravity on one of the side planes, L—Platform length, H—Platform width.

Based on the measured response of the supports of the weighing scale platform, the position of the centre of gravity f_ij_ on four planes π1–π4, in relation to the system with a start point at the weighing scale platform W1 (Fig 2), was determined. Based on the position, the coordinates of the position of the centre of gravity on one plane, parallel to the floor, were determined using Eqs (1) and (2).

$$x=\frac{-\frac{L_{2}f_{12}}{f_{43}-f_{12}}-f_{14}}{\left(\frac{f_{23}-f_{14}}{L_{1}}\right)-\left(\frac{L_{2}}{f_{43}-f_{12}}\right)}$$ (1)

$$y=\frac{\left(\frac{f_{23}-f_{14}}{L_{1}}\right)\left(\frac{L_{2}f_{12}}{f_{43}-f_{12}}\right)-\left(\frac{f_{23}-f_{14}}{L_{1}}\right)f_{14}}{\left(\frac{f_{23}-f_{14}}{L_{1}}\right)-\left(\frac{L_{2}}{f_{43}-f_{12}}\right)}+f_{14}$$ (2)

**Fig 3:**
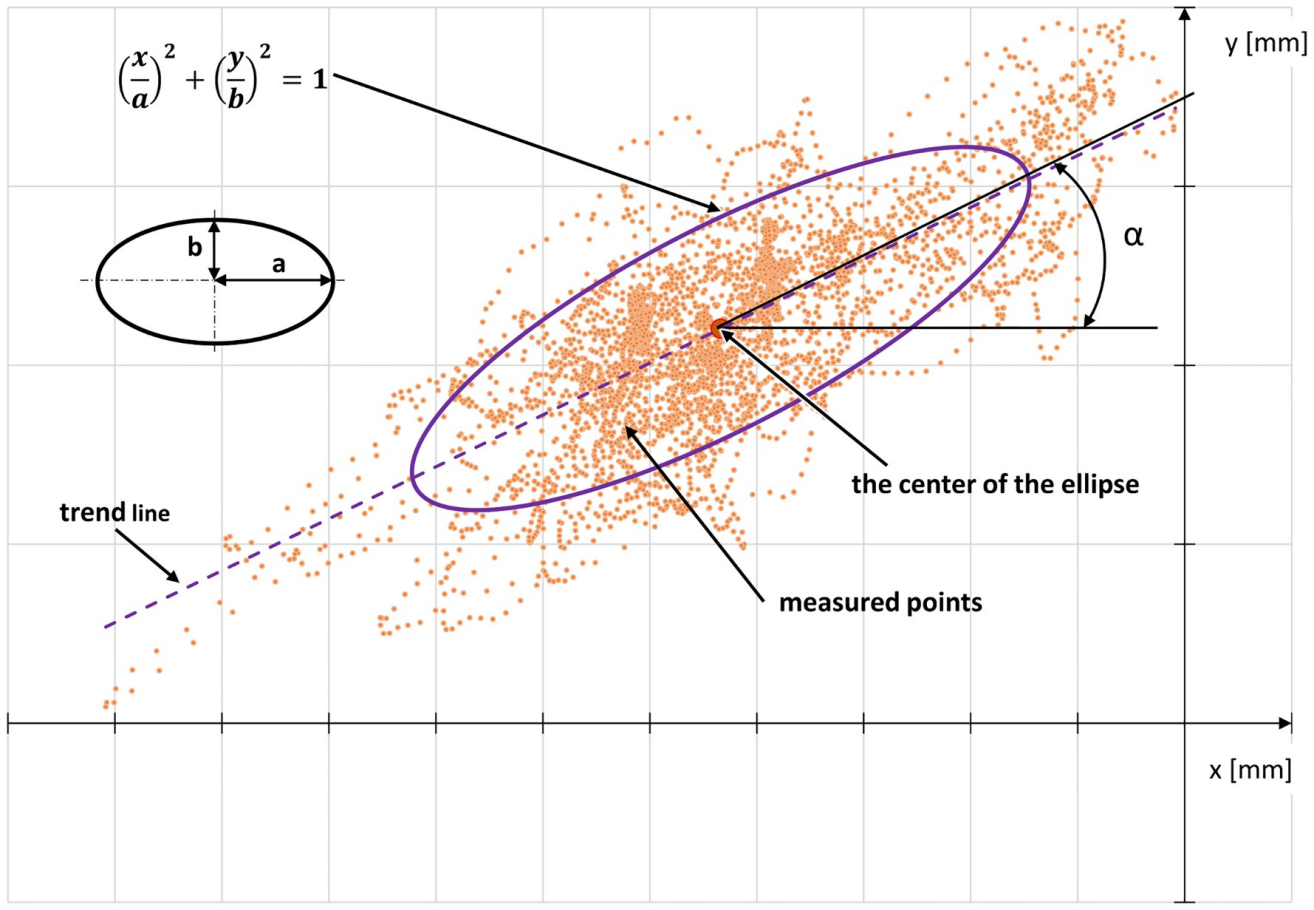
Diagram representing the ellipse used to determine the variability of the position of the centre of gravity of the human body based on the measured points of the position of the centre of gravity, where α—Inclination of the axis of the ellipse, a—Semimajor axis, b—Semiminor axis.

The centre of gravity was determined every 0.01 seconds in each test, giving a set of points. The set of points representing the position of the centre of gravity of the human body in time was replaced with an ellipse (Fig 3), with the central point in the plot being an average of the measured coordinates of the position of the centre of gravity, and the inclination angle α of the semi-major axis of the ellipse corresponded to the slope of a trend line for the measured points.

The dimensions of the ellipse that replaced the set of points of the measured centre of gravity of the human body were determined in such a way that at least 75% of the measured points were in the area drawn by the ellipse. Knowing that the measured coordinates of the centre of gravity need not be characterized by a normal distribution, the Chebyshev inequality was used in which for k = 2 a maximum of 25% of the measured points was rejected. On this basis, the dimensions of the semi-axes of the ellipse a and b were determined by calculating the standard deviation σ_x_ and σ_y_ multiplied by the adopted factor k according to Eqs (3) and (4).

$$a=k\sigma_{x}$$ (3)

$$b=k\sigma_{y}$$ (4)

**Fig 4:**
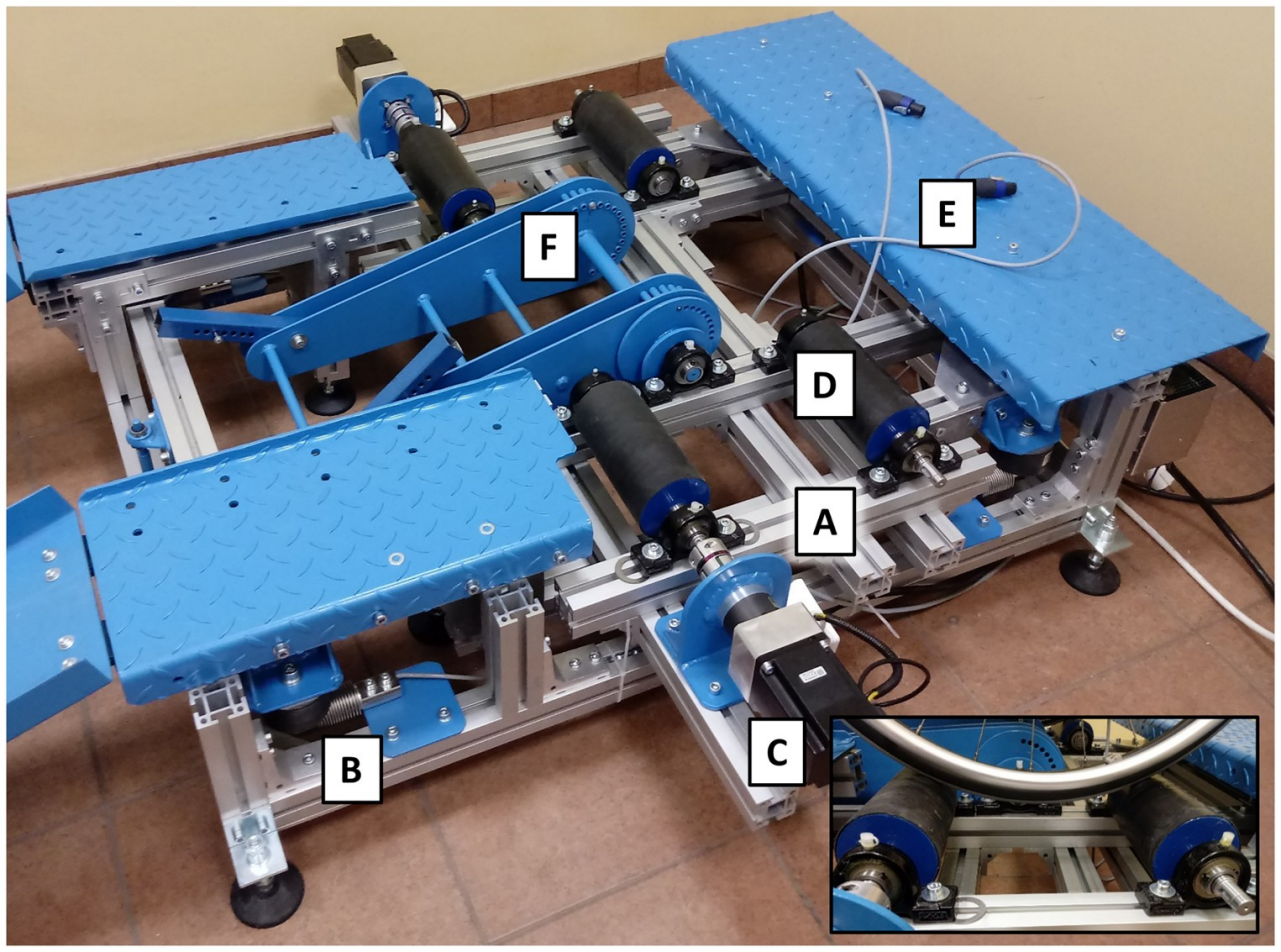
Test stand for simulating operating conditions and measuring dynamic parameters of a wheelchair, see description in the article, where A—Weighing scale platform, B—Strain gauges, C—Electric motors, D—Traction rollers, E—Incremental encoders, F—Tilting arm.

A test stand for simulating operating conditions and measuring dynamic parameters of a wheelchair [17] was used in the tests (Fig 4). The basic component of the test stand is a weighing scale platform (Fig 4A) with four strain gauges (Fig 4B). The rear drive wheels are supported on the traction rollers (Fig 4D) coupled with two electric motors (Fig 4C) simulating the resistance to motion. The front section of the wheelchair is connected to an arm tilting the wheelchair (Fig 4F). Kinematic parameters were measured using two incremental encoders (Fig 4E) coupled with the rear drive wheels.

**Fig 5:**
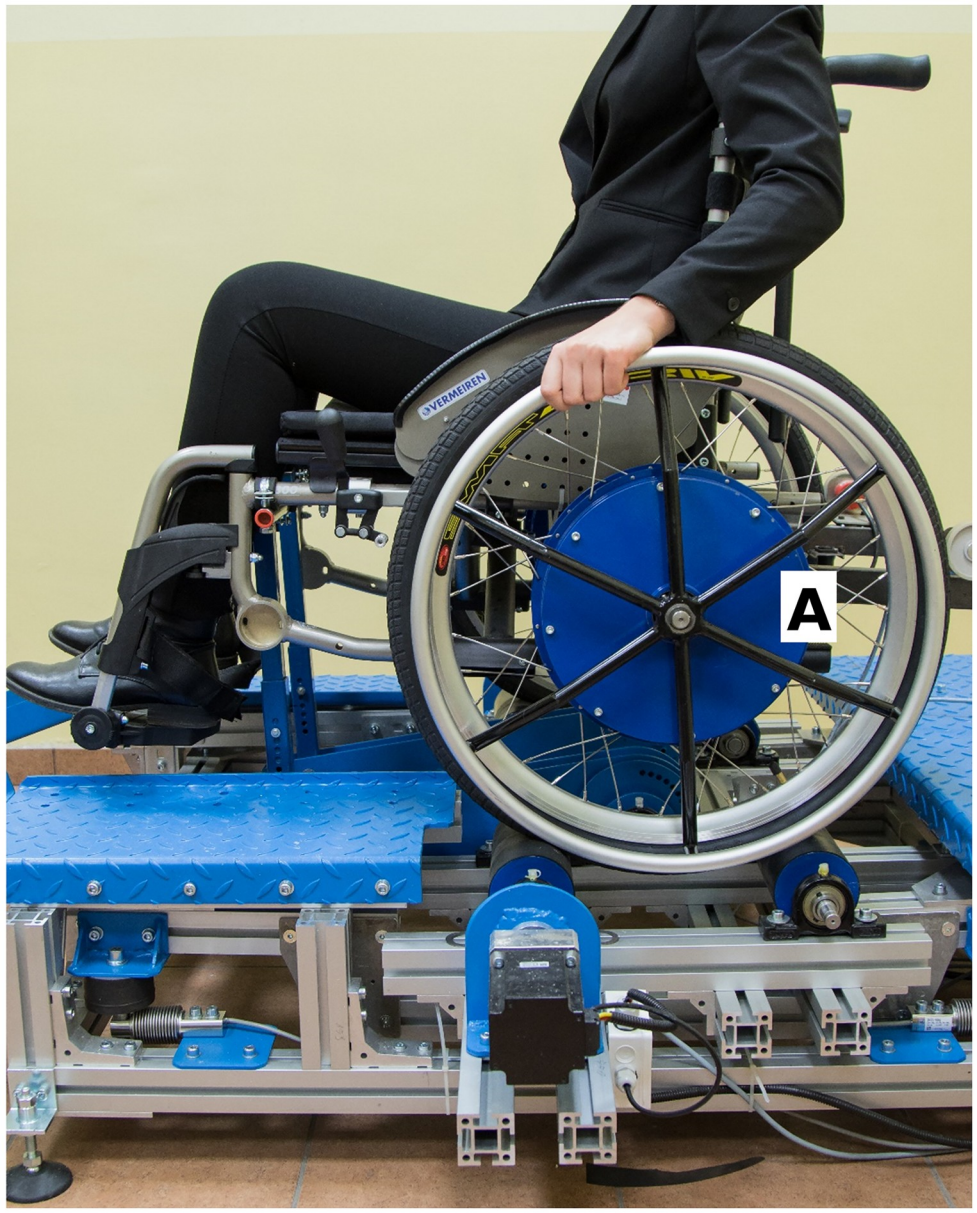
Tested wheelchair at the test stand, where A—Multispeed hub gearbox.

A manually propelled wheelchair with multi-gear transmission (3 gears) was tested [18] (Fig 5). The wheelchair was propelled by the operator using three constant propulsion frequencies at three different wheelchair tilt angles and three gear ratios (Table 1). Propulsion frequency was controlled by a metronome. The operator’s task was to execute individual propulsion phases according to the rhythm set by this device. The drive wheels were loaded with the same constant anti-torque for all tests. A total of 27 tests were carried out. During the test, the position of the centre of gravity of the wheelchair was measured in constant time intervals.

**Table 1:** Test configurations: Velocity v_i_, wheelchair tilt angle k_i_ and gear ratio p_i_.

index	Frequency of pushing v_i_ [push per minute]	Angle of inclination k_i_ [°]	Gear ratio p_i_ [–]
**1**	40	0	1.96
**2**	45	1.5	1
**3**	50	5.4	0.51

The results were compared with only one of the operational parameters of the wheelchair variable. For example, changes in the propulsion frequency were measured at a constant wheelchair tilt angle and constant gear ratio. The result of the test procedure was 27 tests, for which the region of variability of the position of the centre of gravity of the human body was determined in relation to the gear ratio, propulsion frequency and wheelchair tilt angle. To recognise the defined goal as accomplished, it took one person, because the research was qualitative not quantitative. The key task was to establish whether the change in the constructed devices (research stand and drive transmission with epicyclic gear) results in a measurable change of the position of the centre of gravity. Currently, the relations between the quantitative factors of separate parameters are being examined, such as muscular effort and the position of the centre of gravity. It is, however, beyond the scope of this research, which has a finite character. To enable statistical analysis of the collected results, it was decided to use a large number of propulsion cycles.

One female subject, aged 25 years, with a body weight of 61 kg and the BMI value of 20,4 kg/m^2^ was examined. The examined person is a student at the University of Physical Education in Poznań, and volunteered as a test subject. She confirmed the voluntary willingness to participate in the study with the signature on the consent form and read the patient information form. The research has been positively evaluated by Bioethical Commission at the Karol Marcinkowski Medical University in Poznań Poland, Resolution No. 1100/16 of 10 November 2016, under the guidance of Prof. MD Chęciński P. for the research team led by Ph.D. Wieczorek B. The authors obtained written consent of the examined person for the publication of research results with her participation. The data was presented in such a way as to ensure her complete anonymity. The set of registered results describes only one patient. The analysis does not concern a larger number of people, and therefore they are not categorised. On this basis, it should be stated that there are no ethical concerns.

## Results

During the tests, the effects of the selected operational parameters of the wheelchair on the region of variability of the position of the centre of gravity of the human body in the sagittal plane was determined. Variability of the position of the centre of gravity was defined with ellipses covering 80–90% of all measured points of the position of the centre of gravity of the human body for a single test, in which the wheelchair operator carried out 30 propulsion cycles. The test aimed to analyse the effects of the following parameters:

- gear ratio at constant propulsion frequency and constant wheelchair tilt angle;
- propulsion frequency at constant gear ratio and constant wheelchair tilt angle;
- wheelchair tilt angle at constant propulsion frequency and constant gear ratio.

**Fig 6:**
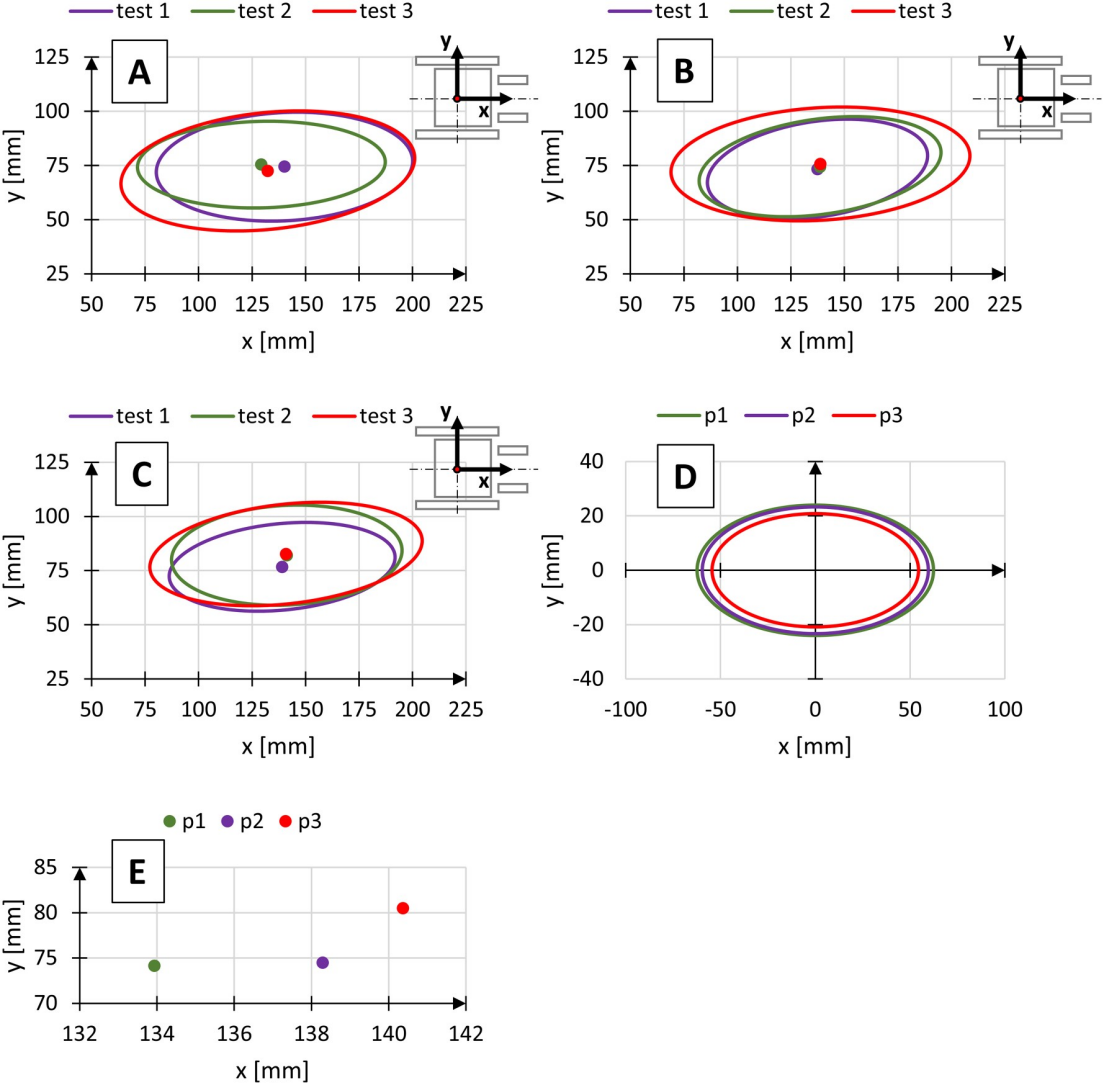
Diagrams of regions of the position of the centre of gravity for three gears ratios p1–i = 1.96 (A), p2–i = 1 (B), p3–i = 0.51 (C), medium regions of the position of the centre of gravity (D) and location of medium centre of gravity (E), where test—Independent measuring test.

**Fig 7:**
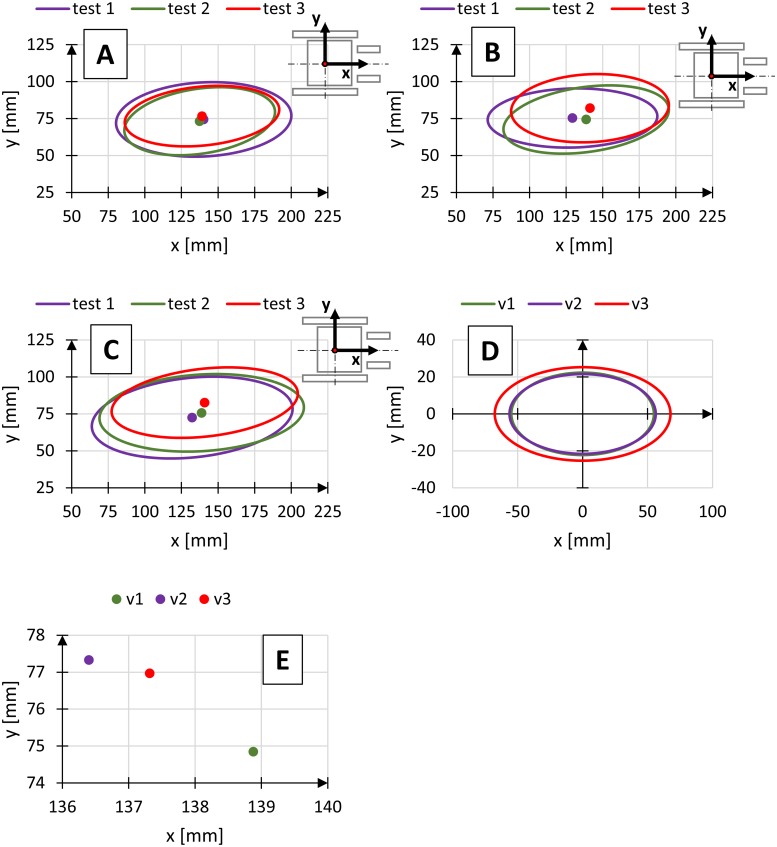
Diagrams of regions of the position of the centre of gravity for three propulsion velocities v1–v = 40 ppm (A), v2–v = 45 ppm (B), v3–v = 50 ppm (C), medium regions of the position of the centre of gravity (D) and location of medium centre of gravity (E), where test—Independent measuring test.

**Fig 8:**
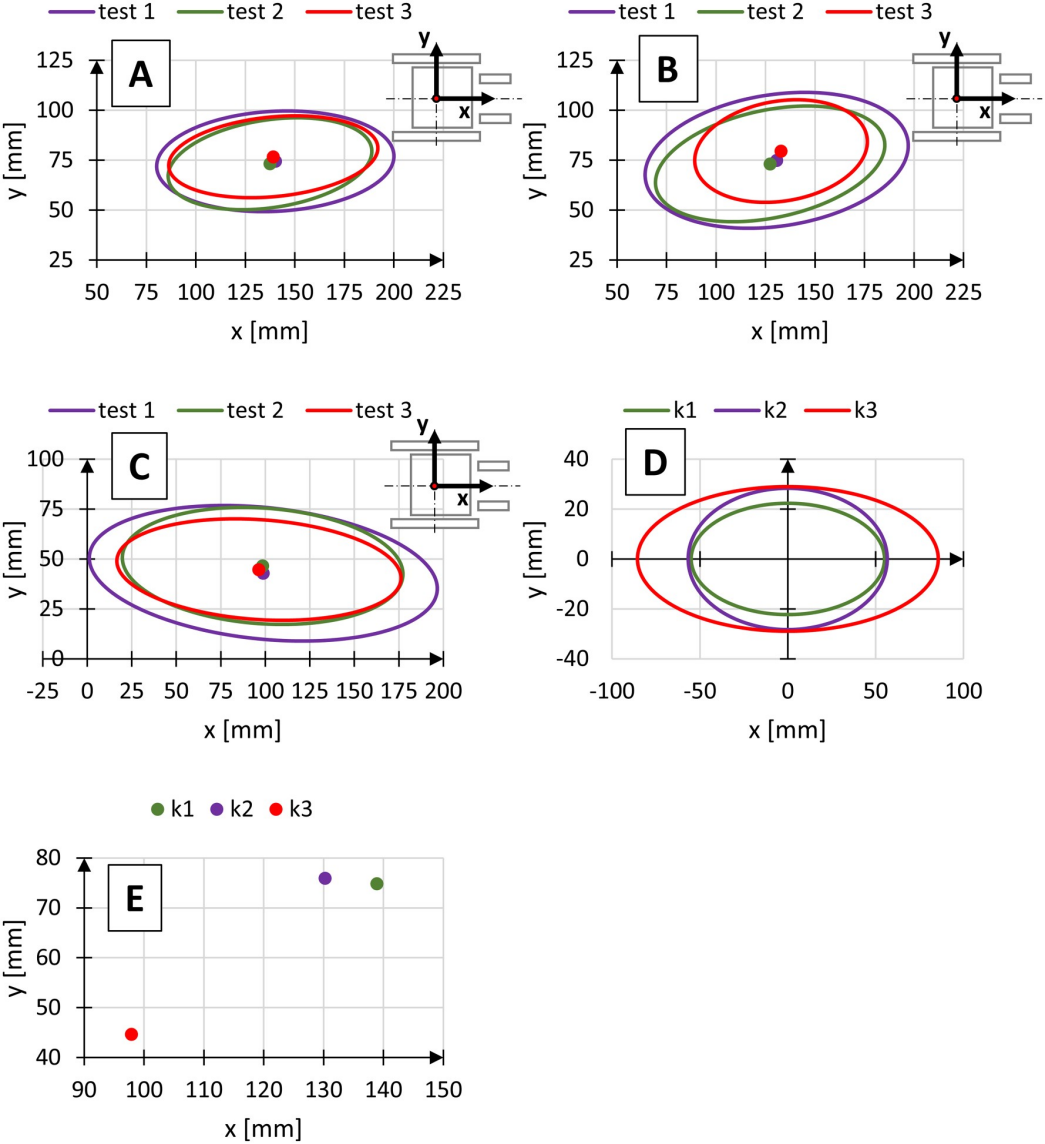
Diagrams of three regions of the position of the centre of gravity at three wheelchair tilt angle k1–β = 0° (A), k2–β = 1.5° (B), k3–β = 5.4° (C), medium regions of the position of the centre of gravity (D) and location of medium centre of gravity (E), where test—Independent measuring test.

The central point on the axis of rotation of the rear wheels was selected as a starting point of the system of coordinates representing the changes in the position of the centre of gravity of the human body. The ellipses were drawn in the sagittal plane and defined using three parameters: inclination angle α of the semi-major axis a in relation to the axis x, corresponding to the travel direction, semi-major axis a defining the length of the ellipse in the direction corresponding to the travel direction, and semi-major axis b defining the width of the ellipse, perpendicular to the semi-major axis a. Charts showing the differences in registered positions of the centre of gravity for different conditions (Figs 6–8).

Tables 2–4 show the parameters of the ellipses describing the region of variability of the position of the centre of gravity for selected wheelchair operational parameters.

**Table 2:** Parameters of the ellipses describing the region of variability of the position of the centre of gravity for selected operational parameters of the wheelchair and three gear ratios, where i—Gearbox ratio, test—Independent measuring test, α—Inclination of the axis of the ellipse, a—Horizontal axis of the ellipse, b—Vertical axis of the ellipse, M—Average value, SD—Standard deviation, x_CG_—Average position of the centre of gravity on x-axis, y_CG_—Average position of the centre of gravity on y-axis, ρ—The number of measured points inside the ellipse.

	**p1—i = 1.96**				**p2—i = 1.00**				**p3—i = 0.51**			
	α	a	b	ρ	α	a	b	ρ	α	a	b	ρ
	[°]	[mm]	[mm]	[%]	[°]	[mm]	[mm]	[%]	[°]	[mm]	[mm]	[%]
**test 1**	2.94	59.99	24.99	84.2	0.06	42.00	19.00	87.30	10.83	70.00	26.00	85.70
**test 2**	1.37	57.96	19.95	84.9	8.74	41.00	18.00	86.00	11.19	55.00	20.00	84.90
**test 3**	5.79	68.95	26.95	86.4	2.55	35.00	18.00	85.70	10.41	39.00	22.00	85.10
**M**	3.37	62.30	23.96	---	3.78	39.33	18.33	---	10.81	54.67	22.67	---
**SD**	2.24	5.85	3.61	---	4.47	3.79	0.58	---	0.39	15.50	3.06	---
**Center of gravity**			**x**_**CG**_	**y**_**CG**_			**x**_**CG**_	**y**_**CG**_			**x**_**CG**_	**y**_**CG**_
			[mm]	[mm]			[mm]	[mm]			[mm]	[mm]
	**test 1**		140.15	74.50	**test 1**		137.43	73.31	**test 1**		139.06	76.74
	**test 2**		129.32	75.43	**test 2**		138.66	74.45	**test 2**		141.22	82.11
	**test 3**		132.32	72.51	**test 3**		138.79	75.72	**test 3**		140.83	82.68
**M**	---		133.93	74.14	---		138.29	74.49	---		140.37	80.51
**SD**	---		5.59	1.49	---		0.75	1.20	---		1.15	3.28

**Table 3:** Parameters of the ellipse describing the region of variability of the position of the centre of gravity for the selected operational parameters of the wheelchair and three propulsion velocities, where v—Frequency of push, test—Independent measuring test, α—Inclination of the axis of the ellipse, a—Horizontal axis of the ellipse, b—Vertical axis of the ellipse, M—Average value, SD—Standard deviation, x_CG_—Average position of the centre of gravity on x-axis, y_CG_—Average position of the centre of gravity on y-axis, ρ—The number of measured points inside the ellipse.

	**v1–40 ppm**				**v2–45 ppm**				**v3–50 ppm**			
	α	a	b	ρ	α	a	b	ρ	α	a	b	ρ
	[°]	[mm]	[mm]	[%]	[°]	[mm]	[mm]	[%]	[°]	[mm]	[mm]	[%]
**test 1**	2.94	59.99	24.99	84.2	1.37	57.96	19.95	84.90	5.79	68.95	26.95	86.40
**test 2**	8.42	51.94	21.98	87.3	7.78	56.98	21.98	86.00	3.54	70.00	25.97	85.70
**test 3**	5.52	52.99	19.95	85.7	2.85	53.97	22.96	84.90	6.25	63.98	22.96	85.10
**M**	5.63	54.97	22.31	---	4.00	56.30	21.63	---	5.19	67.64	25.29	---
**SD**	2.74	4.38	2.54	---	3.36	2.08	1.54	---	1.45	3.22	2.08	---
**Center of gravity**			**x**_**CG**_	**y**_**CG**_			**x**_**CG**_	**y**_**CG**_			**x**_**CG**_	**y**_**CG**_
			[mm]	[mm]			[mm]	[mm]			[mm]	[mm]
	**test 1**		140.15	74.50	**test 1**		129.32	75.43	**test 1**		132.32	72.51
	**test 2**		137.43	73.31	**test 2**		138.66	74.45	**test 2**		138.79	75.72
	**test 3**		139.06	76.74	**test 3**		141.22	82.11	**test 3**		140.83	82.68
**M**	---		138.88	74.85	---		136.40	77.33	---		137.32	76.97
**SD**	---		1.37	1.74	---		6.26	4.17	---		4.45	5.20

**Table 4:** Parameters of the ellipses describing the region of variability of the position of the centre of gravity for selected operational parameters of the wheelchairs and three wheelchair tilt angles β, where test—Independent measuring test, α—Inclination of the axis of the ellipse, a—Horizontal axis of the ellipse, b—Vertical axis of the ellipse, M—Average value, SD—Standard deviation, x_CG_—Average position of the centre of gravity on x-axis, y_CG_—Average position of the centre of gravity on y-axis, ρ—The number of measured points inside the ellipse.

	**β - 0°**				**β– 1.5°**				**β– 5.4°**			
	α	a	b	ρ	α	a	b	ρ	α	a	b	ρ
	[°]	[mm]	[mm]	[%]	[°]	[mm]	[mm]	[%]	[°]	[mm]	[mm]	[%]
**test 1**	2.94	59.99	24.99	84.2	8.28	66.99	32.97	86.20	-5.06	98	32.97	87.60
**test 2**	8.42	51.94	21.98	87.3	11.67	58.94	26.95	87.90	-3.45	78.96	28.98	86.00
**test 3**	5.52	52.99	19.95	85.7	9.08	43.96	24.99	87.00	-3.38	79.94	24.99	86.20
**M**	5.63	54.97	22.31	---	9.68	56.63	28.30	---	-3.96	85.63	28.98	---
**SD**	2.74	4.38	2.54	---	1.77	11.69	4.16	---	0.95	10.72	3.99	---
**Center of gravity**			**x**_**CG**_	**y**_**CG**_			**x**_**CG**_	**y**_**CG**_			**x**_**CG**_	**y**_**CG**_
			[mm]	[mm]			[mm]	[mm]			[mm]	[mm]
	**test 1**		140.15	74.50	**test 1**		130.64	74.95	**test 1**		98.85	42.83
	**test 2**		137.43	73.31	**test 2**		127.29	73.19	**test 2**		98.45	46.54
	**test 3**		139.06	76.74	**test 3**		132.85	79.61	**test 3**		96.39	44.70
**M**	---		138.88	74.85	---		130.26	75.92	---		97.90	44.69
**SD**	---		1.37	1.74	---		2.80	3.32	---		1.32	1.85

## Discussion

Changes in the gear ratio of the propulsion system affected the load on the muscular system of the upper limbs and the shoulder girdle of the operator. The higher the gear ratio of the multi-gear transmission of the tested wheelchair, the higher the load on the muscular system. Analysis of the variability of the region of the position of the centre of gravity depending on the gear ratio for different operating conditions (Fig 6, Table 2) shows an slight increase in ellipse size with an increase in gear ratio. Semi-major axis a of the ellipse describes the variability of the position of the centre of gravity changes to the highest degree. Dimension changes for the semi-axis a are 7.6 mm while for the semi-axis b are 1.3 mm.

The change in load affects the kinematics of the upper limb propelling the pushrim [4]. The load acting on the propulsion system affects the motion of the upper limbs and torso and changes the dimensions of the region of variability of the position of the centre of gravity of the human body. In the tests, an increase in the propulsion force [19] resulted in more chaotic movements of the upper limb and elongation of the hand motion path to the front of the wheelchair [20]. It resulted in an increase in ellipse dimensions, with an increase in the gear ratio of the wheelchair’s propulsion system.

Analyses of the results of the dimensions of the regions of variability of the centre of gravity at different gear ratios show that the dimensions of the ellipses differ slightly despite a growing tendency (Table 2). An increase in semi-major axis a, in relation to semi-major axis b with an increase in gear ratio, can be observed for all operating conditions.

Another variable parameter was the propulsion frequency expressed as the number of push motions per minute. The wheelchair velocity was controlled by changing the propulsion frequency. Based on the measured position of the centre of gravity of the human body, the ellipses describing the variability of the centre of gravity at three propulsion frequencies at constant wheelchair tilt angle were determined (Fig 7, Table 3). Analysis of the results shows that with an increase in the propulsion frequency, semi-major axis a of the ellipse lengthens, which translates into an increase in the region of variability of the position of the centre of gravity of the human body. The results also show that the propulsion frequency increases the scatter of the points of the position of the centre of gravity along the travel direction (axis x), measured dimensional difference for extreme propulsion frequency values was 12.7 mm. It results from an increase in the torso flexion contribution and changes in the hand motion path [20], with an increase in the propulsion frequency. The tests show that changes in the propulsion frequency mainly affect the hand motion path [4, 20, 21]. It changes the kinematics of the upper limb and the distribution of the position of the centre of gravity of the human body.

Analysis of the changes in the frequency of wheelchair propulsion does not show a significant effect on ellipse inclination angle and distribution of the points of the position of the centre of gravity of the human body in the direction lateral to the travel direction (axis y). For extreme values of the propulsion frequency, the semi-axis dimension b differed by 3.0 mm. It can be explained by the biomechanics of wheelchair propulsion, in which an increase in propulsion frequency translates to changes in the shift of the human body segments in the direction corresponding to the travel direction only (axis x).

In reference to the actual operating conditions, the wheelchair tilts as a result of uneven terrain. To reflect those conditions in the tests, the wheelchair’s frame was tilted by lifting the front castor wheels to determine the effect of wheelchair tilt angle β on the distribution of the position of the centre of gravity of the human body at constant propulsion frequency and constant gear ratio (Fig 8, Table 4).

Changes in the wheelchair tilt angle β resulted in a shift in the ellipse position in the direction of the axis of rotation of the rear wheelchair wheels, with an increase in the tilt angle. It is caused by the body shifting closer to the wheel axis as a result of the wheelchair seat tilt.

Analysis of the dimensions of semi-major axis a and b of the ellipses defining the variability of the position of the centre of gravity of the human body shows that the dimensions increase with an increase in the tilt angle. The results show that the most extensive changes in the position of the centre of gravity of the human body can be observed for the tilt angle β = 5.4°, irrespective of the propulsion frequency and gear ratio. An increase in the surface area of the ellipses is due to an increase in torso flexion and an increase in the wheelchair tilt angle. High increase in semi-axis a dimension (of 30.7 mm), resulted from high torso flexion angles during wheelchair propulsion at high seat [22] tilt angle.

Comparison of the average dimensions of ellipses shows that the biggest difference between the dimensions of the region of variability of the position of the centre of gravity of the human body can be observed for changes in the wheelchair tilt angle. For the semi-axis a the increase was 30.7 mm and for the semi-axis b 6.8 mm. Also, the propulsion frequency affects the dimensions of the ellipses. In this case, the semi-axis dimension a increased by 12.7 mm as the propulsion frequency increased. The analysis of the effect of the gear ratio on the region of variability of the position of the centre of gravity of the human body shows a minor effect of this parameter. In the case of semi-axis a the difference in dimensions was 7.6 mm and for the semi-axis b the dimensional difference was 1.3 mm.

Analysis of the changes in the dimensions of the semi-axles shows that any changes in operating parameters affect major on the dimensions of semi-axis a and the ellipse inclination angle in relation to axis x. The tested configurations show that the operating parameters affect the dimension of semi-axis b of the ellipse defining the distribution of the centre of gravity in relation to the left and right wheelchair wheel to a negligible degree.

## Summary

The tests verified the effects of the operational parameters of a wheelchair on the position of the centre of gravity of the body of an operator propelling a manual wheelchair. During the test, the gear ratio of the propulsion drive, propulsion frequency and wheelchair tilt angle were variable. For all tests, an increase in aforementioned parameters resulted in any of the parameters resulted in an increase in effort required by the operator. The effort was compensated by the wheelchair operator changing their body kinematics, which translated into changes in the dimensions of the regions of variability of the position of the centre of gravity defined with ellipses. It can be assumed that an increase in wheelchair resistance to motion [23] translates to an increase in the variability region of the position of the centre of gravity of the human body.

The results show that the position and dimensions of the variability region of the position of the centre of gravity are affected by the wheelchair tilt angle. The results show that an increase in the wheelchair tilt angle increases the variability region of the position of the centre of gravity in the direction corresponding to the travel direction. The second operational parameter affecting the region of variability of the position of the centre of gravity is the wheelchair propulsion frequency. In this case, with an increase in the propulsion frequency, the region of variability of the position of the centre of gravity also increases. Variability of the dimensions of the ellipses defining the position of the centre of gravity is least affected by changes in the gear ratio.

The increase in the region of variability of the position of the centre of gravity of the human body as a result of the increase of the wheelchair tilt angle results from changes in the position of the human body in relation to the axis of rotation of the drive wheel and the wheelchair seat inclination [22]. It increases the torso and shoulder girdle movement contribution, which due to their mass, significantly increase the longitudinal dimension of the ellipse corresponding to the travel direction. For the increase in propulsion frequency, an increase in the region of variability of the position of the centre of gravity is due to an increase in the range of motion of the upper limb. The observations show that an increase in the propulsion frequency also affects the torso flexion and extends the ellipse in the longitudinal direction, corresponding to the travel direction.

A common relationship for all the operational parameters was an increase in the longitudinal dimension of the ellipses corresponding to the travel direction with only slight changes in the lateral dimensions. The ellipse inclination angle for each test depends on the uniformity of wheelchair propulsion and torso flexion. The observations show that the inclination angle indicates which side of the wheelchair is loaded at a time. Positive α angle indicates the left and negative α angle indicates the right side of the wheelchair. The position of the ellipse in the horizontal plane depends on the seating position of the operator and the position of the body in relation to the sagittal plane. The test results were in the 1st quarter of the reference system, indicating an asymmetric position of the body in relation to the seat and shift in the direction of the left drive wheel.

The tests represent the effects of operational parameters on the distribution of the position of the centre of gravity in dynamic conditions. Since the position of the centre of gravity of the wheelchair is constant, the position of the centre of gravity of the entire system is affected by the variable position of the centre of gravity of the human body. The position of the centre of gravity of the human-wheelchair system is the result of the kinematics of the human body propelling the manual wheelchair. The design of innovative and advanced wheelchairs requires thorough knowledge of the biomechanical parameters affecting the wheelchair operation. The position of the centre of gravity is a major biomechanical parameter affecting wheelchair stability [24, 25]. Stability is a key safety factor. The position of the centre of gravity also affects changes in resistance to motion and thus the muscular activity of the upper limbs and shoulder girdle. Determining the position of the centre of gravity of the human body in the horizontal plane is important. It allows to define the place of load application to the wheelchair structure (the mass force that the support frame has to withstand). The innovation of the described method is to perform measurements in dynamic conditions, which allows to significantly refine the load values assumed in calculations regarding the strength of the structure and its stability. The novelty of the results obtained in the presented method is their determination based on various parameters, in dynamic conditions. The realised experiments represent a selected part of the ongoing research. In order to consider them as complete, it is necessary to measure the position of the centre of gravity also on the vertical axis. This is necessary in order to determine the dynamic stability. This measurement is carried out within the described research project, but with the use of another method not included in this article.

The presented test results allow the assessment of the change of the position of the centre of gravity depending on the movement conditions of the manually operated wheelchair. Therefore, it is possible to determine the pressure values (reaction forces) to each wheelchair wheel, among others. It is a crucial parameter which determines, for instance, the torque required to move a wheelchair. This research is unique also due to the use of an original research stand and innovative transmission of manual drive to the wheelchair wheels, which was submitted as a patent application [12, 17]. Work related to the development of innovative designs of manual wheelchairs is important because the physical activity associated with the propelling of the wheelchair enriches the rehabilitation process. Wheelchairs equipped with electric drive do not have this advantage. The developed constructional solutions and the results of the conducted research remain an added value in the field of both theoretical research and practical design guidelines for wheelchairs and their drive systems. Therefore, it broadens the scope of the design of adjusting the wheelchair to individual needs of disabled people.

## Conclusions

According to the research and analysis, it can be established that the tilt angle, gear ratio of the propulsion system and propulsion frequency of a wheelchair influence the position of the centre of gravity. The biggest observed change is the result of the largest wheelchair tilt angle. Most likely, it is a result of the increased effort that the operator had to put in when propelling the wheelchair. It directly influenced the position of the centre of gravity during the test. It can be concluded that the research stand used, enables to examine in a measurable way the influence of individual parameters on the position of the centre of gravity. Establishing the quantitative influence on this parameter is the aim for further research.

## Supporting information

S1 File (RAR)
